# An Unusual Case of Hemorrhagic Pleuropericarditis After COVID-19 Vaccination

**DOI:** 10.7759/cureus.24828

**Published:** 2022-05-08

**Authors:** Aneeqa Javed, Yelizaveta Medina, Julia Tian, Muhammad Junaid Alvi, Syeda Sahra, Geurys Rojas-Marte

**Affiliations:** 1 Internal Medicine, Northwell Health, Staten Island, USA; 2 Cardiology, Northwell Health, Staten Island, USA; 3 Critical Care Medicine, Mount Sinai Hospital, Manhattan, USA

**Keywords:** hemorrhagic, vaccine, covid-19 vaccine complication, covid-19 vaccine, pericardial diseases, covid 19

## Abstract

As coronavirus disease 2019 (COVID-19) vaccines are being increasingly administered worldwide, subsequent side effects, such as myocarditis, pericarditis, and myopericarditis, are becoming increasingly more common. Our case describes a 64-year-old male who developed chest pain and shortness of breath one week after receiving the Moderna (Cambridge, Massachusetts) COVID-19 mRNA vaccine. He was found to have a large, left-sided pleural effusion and a small pericardial effusion. The patient underwent thoracentesis and video-assisted thoracoscopic procedure with chest tube placement, which drained bloody pleural and pericardial fluid. He was treated with a course of colchicine. Subsequent imaging revealed the resolution of pericardial and pleural effusions, along with the resolution of symptoms.

## Introduction

Vaccines are our frontline defense in the fight against coronavirus disease 2019 (COVID-19). Numerous minor side effects have been reported, which are managed conservatively; however, rare side effects, including myocarditis, pericarditis, and myopericarditis, have come to light between December 2020 and June 2021 after the Food and Drug Administration (FDA) approved Emergency Use Authorizations (EUAs) for the Pfizer-BioNTech (New York NY) COVID-19 and Moderna (Cambridge, Massachusetts) COVID-19 mRNA vaccines [1-3]. Here we present a case showing the association of hemorrhagic pericardial effusion with the COVID-19 mRNA vaccine. To the best of our knowledge, no such case has been reported to date.

## Case presentation

A 64-year-old male presented to our emergency department (ED) with retrosternal chest pain associated with shortness of breath. He had a past medical history of nonobstructive coronary artery disease, permanent pacemaker secondary to atrioventricular (AV) block, rheumatic heart disease, and a small right ventricular apical aneurysm of unknown etiology. His home medications included aspirin, losartan, hydrochlorothiazide, and atorvastatin. He was not on any oral anticoagulation. His chest pain and shortness of breath started one week ago after he received the second dose of the mRNA COVID-19 vaccine Moderna. He went to the ED and investigations at that time included a coronary CT angiography (CCTA) that showed patent left main and left anterior descending (LAD) arteries, unchanged appearance of the known right ventricular apical pseudoaneurysm, and trace left pleural effusion. He was discharged and managed by his cardiologist for suspected pericarditis. Over the next few days, his symptoms worsened so he returned to our ED. He was found to be hypoxemic on the exam and placed on 2 liters per minute of supplementary oxygen via nasal cannula. On auscultation of his chest, he had decreased breath sounds on the left side and initial blood work showed no abnormalities with normal kidney and liver function tests (Table 1).

**Table 1:** Bloodwork on admission including hematology panel and comprehensive metabolic panel eGFR: estimated glomerular filtration rate

WBC Count	8.14 K/uL
Hemoglobin	13.7 g/dL
Platelet Count	261 K/uL
Sodium, Serum	139 mmol/L
Potassium, Serum	4.5 mmol/L
Creatinine, Serum	0.7 mg/dL
Albumin, Serum	3.5 g/dL
Alkaline Phosphatase, Serum	48 U/L
Aspartate Aminotransferase, Serum	39 U/L
Alanine Aminotransferase, Serum	53 U/L
eGFR	100 mL/min/1.73m^2^)

**Figure 1:**
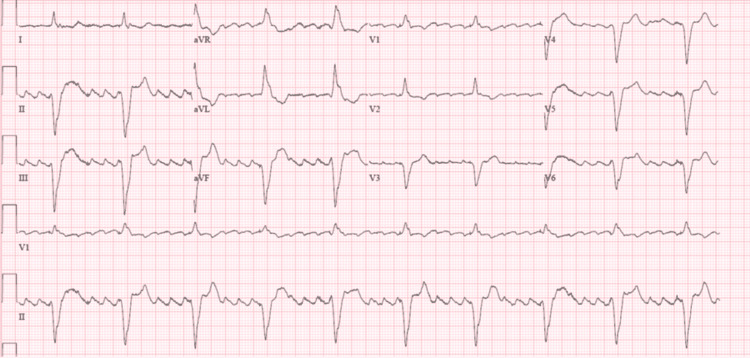
EKG on admission showing atrial fibrillation with a rapid ventricular response

An EKG showed a new-onset, rate-controlled atrial flutter (Figure 1).

**Figure 2:**
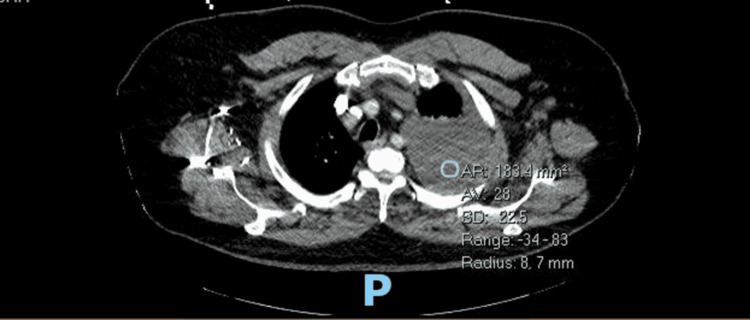
A large, left-sided pleural effusion and a small pericardial effusion seen on CT chest

A CT scan of the chest revealed a large, left-sided pleural effusion and a small pericardial effusion (Figure 2).

**Figure 3:**
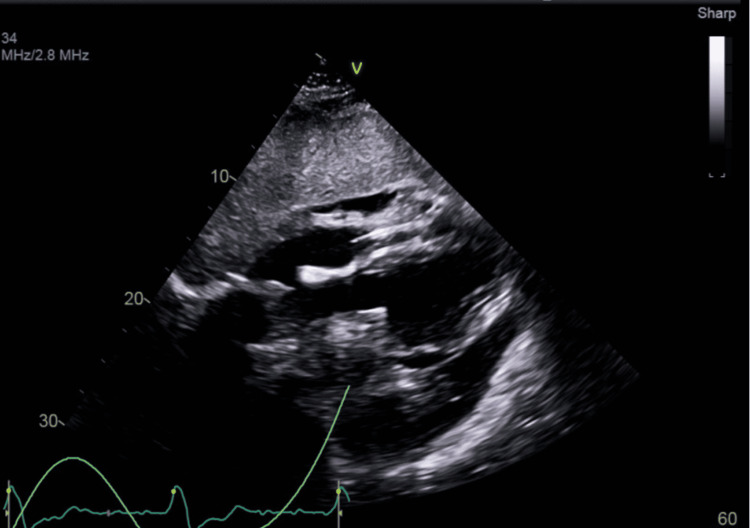
A small pericardial effusion and apparent diastolic collapse of the right ventricle seen on echocardiogram

An echocardiogram showed normal global left ventricular systolic function, small pericardial effusion, and apparent diastolic collapse of the right ventricle (Figure 3).

A thoracentesis was performed and 1200 ml of bloody pleural fluid was drained. Radiographic imaging after the procedure showed decreased left pleural effusion with improved aeration of the left upper lobe and no pneumothorax was seen. His hemoglobin remained stable. He then underwent a video-assisted thoracoscopic procedure with left-sided chest tube placement and another 1000 ml of bloody pleural fluid was drained. No evidence of cardiac tamponade was seen. There was also no RV perforation or sign of a leak from the aneurysm. The chest tube was successfully removed in four days with improvement in the patient’s symptoms. Pleural fluid histochemistry showed bloody exudative fluid and mostly red blood cells (total red blood cell count 696,000/uL, hematocrit 6.96%) with no signs of malignancy. The rheumatological workup including double-stranded DNA antibody, anti-nuclear factor titer, and quantitative rheumatoid factor was negative. The patient had a rapidly accumulating hemorrhagic pleural effusion in the absence of right ventricular (RV) perforation or leakage suggesting inflammatory etiology. He was managed with colchicine, pain medications, and drainage of the pleural effusion. He was discharged with outpatient follow-up with his cardiologist and an outpatient chest X-ray one week later showed resolution of the pleural effusion and complete resolution of his symptoms.

## Discussion

As the number of recipients of COVID-19 vaccination goes up, so does the number of reported side effects. The most common side effects are minor, but rare side effects also come to light. To the best of our knowledge, there have been no reported cases of pericardial effusion after receiving any vaccination. This is the first case of its kind showing an association between a vaccine and pleuropericarditis with pericardial effusion.

The etiology behind large pericardial effusions includes a variety of pathologic processes such as infections, iatrogenic causes, radiation, post-myocardial infarction, autoimmune diseases, renal failure with uremia, and hypothyroidism [4]. Our patient developed pericardial and bilateral pleural effusions after receiving the COVID-19 mRNA vaccine. Cases have been reported showing the association of infections and inflammatory conditions with pleuropericarditis [5-7], but none are vaccine-related.

The pathophysiology behind this process is not well-understood, as there are very limited data. Several mechanisms have been proposed to explain the association between the mRNA vaccine and its cardiac complications such as myocarditis. One of the proposed mechanisms states that this side effect may result from molecular mimicry between the mRNA and some unknown cardiac protein [8]. A recently published, in-depth analysis of a presumed case of myocarditis three days after the second dose of COVID-19 mRNA vaccine in a 52-year-old male concluded that the antibody response in this patient to COVID-19 antigens did not differ from those of vaccinated controls who did not develop any cardiac complications. However, his immune response did differ in an increase in a specific subset of natural killer (NK) cells compared with controls. A causal relationship between these differences and myocarditis could not be established [9].

The incidence of pericarditis is between one and two per million doses of the COVID-19 vaccine. It remains extremely rare. Data collected in the European Economic Area (EEA) suggest that it occurs mostly in young adults, being more common in males [10]. We reviewed some of the literature describing the cardiac complications of mRNA vaccines. Four presented after the first dose in the literature of the 26 cases of myocarditis and pericarditis after receiving the COVID-19 mRNA vaccine. All of them were male, ages 14 to 56, and symptom onset was two to seven days after receiving the COVID-19 vaccine [11-14].

Our patient’s presentation of chest pain and shortness of breath has broad differentials, but imaging and a detailed history helped establish the diagnosis of pleuropericarditis and cardiac tamponade. His symptoms began seven days after receiving the second dose of the COVID-19 vaccine. He was appropriately managed with drainage of the pericardial and pleural fluid. A causal relationship between pleuropericarditis and COVID-19 mRNA vaccine could not be established, and detailed studies of the mechanism behind the immune response to mRNA vaccines are needed.

## Conclusions

With an increasing number of people receiving the COVID-19 mRNA vaccine, an increased number and variety of side effects are coming to light. It is important to correctly identify and diagnose myocarditis, pericarditis, pleuritis, and cardiac tamponade, as timely treatment can save lives. It cannot be proven that the vaccine causes these side effects, and as of November 2021, the Centers for Disease Control and Prevention (CDC) continues to recommend vaccination for all individuals older than age five.
